# A pilot study on the potency of injectable vs. oral moxidectin formulation to suppress strongyle egg excretion in horses at twice lower dose

**DOI:** 10.2478/helm-2025-0018

**Published:** 2025-09-30

**Authors:** M. Šarkūnas, A. Schwahn, K. Suleimanova

**Affiliations:** 1 Department of Veterinary Pathobiology, Veterinary Academy, Lithuanian University of Health Sciences, LT-47181, Kaunas, Lithuania; 2 Department of Natural Sciences, Z. Aldamzhar Kostanay Socio-Technical University, Kostanay, Kazakhstan

**Keywords:** Horse, Moxidectin, Effi cacy, Resistance, Egg reappearance period

## Abstract

The aim of present study was to examine the potency of moxidectin solution (Cydectin 1 %; Zoetis) and commercially available oral moxidectin gel (Equest®; Zoetis) to suppress the excretion of strongyle eggs in horses over a 6-week period. The horses naturally infected with strongyle nematodes (>500 eggs/g of feces) were divided into two comparable groups according age, sex and weight. On day 0 of the study, horses in Group OT (oral treatment; N=5) were dewormed with moxidectin gel (Equest®; Zoetis; 0.4 mg/kg of b.w.) according to the manufacturer’s instructions, and horses in Group IT (intramuscular treatment; N=4) were dewormed with moxidectin injectable solution (Cydectin 1 %; Zoetis; 0.2 mg/kg of b.w.). Stool samples were collected rectally, on day 0, 17, 28, 35, and 42 of the study and examined using a modifi ed McMaster technique (Roepstorff *et al*., 1998) with modifi cations. The mean strongyle egg excretion has signifi cantly decreased in Group IT (P<0.01) and Group OT (P<0.001) on day 17 post treatment as compared to those on day 0. The effi cacy of oral gel (99.43 %) and injectable moxidectin (99.32 %) formulations was therefore high and comparable and no resistance of strongylids to moxidectin was recorded. On day 35 (P<0.001) and 42 (P<0.05) post treatment, the mean effi cacy was signifi cantly higher in Group IT as compared to Group OT. The present study contributes to the existing knowledge and providing more information on the use of injectable MOX solution for the treatment of strongylid infection in horses.

## Introduction

Horses that graze contaminated pastures and are not treated with anthelmintics can be infected with strongylids of several species. The most common nematode species in equines are cyathostomins that belong to the group of small strongyles. Domestic horses can be infected with more than 40 different species of cyathostomins (Kuz’mina, 2012); while more than 10 – 20 species can simultaneously infect one horse (Reinemeyer *et al*., 1984; Chapman *et al*., 2003; Kuzmina, 2012, Kuzmina *et al*., 2016; Bellaw and Nielsen, 2020). In temperate climates the ingested L3 larvae of cyathostomins encyst in the wall of the large intestine over the autumn/winter months and can consist a large part of parasitic burden in horses (Dowdal *et al*., 2002). The small strongyles can cause severe diarrhea, weight loss, colic, edema or fatal colitis when the large numbers of larvae re-emerge from the intestinal mucosa (Giles *et al*., 1985).

Most of adult nematodes in horses are susceptible to nematocidal anthelmintics, however the encysted immature stages of cyathostomins cannot be affected effectively by most of anthelmintics. Moxidectin (MOX) is one of macrocyclic lactones (ML’s) characterized by the broad-spectrum activity against nematodes in a wide range of animal species including horses. It has been demonstrated to have excellent activity against equine nematodes (Lyons *et al*., 1992). It was shown that MOX is superior to other ML’s due to its residual efficacy against nematodes and greater efficacy against inhibited stages of cyathostomins (Monahan *et al*., 1996). The activity of MOX 2 % oral gel at the dose of 0.4 mg/kg of body weight (b.w.), was fully effective in the treatment of *Trichostrongylus axei, Triodontophorus* spp., *Oxyuris equi* L5 stages, and cyathostomins (adult and L5 stages); 99 % against *O. equi* L4 and 92 % to treat *Strongylus edentatus* L5 stages and *Gasterophylus intestinalis* in ponies (Dorchies *et al*., 1998).

Moxidectin was first introduced in 1989 in Argentina as injectable formulation for cattle (Cobb & Boeckh, 2009), and it is currently licensed as an injectable solution (1 %), and as a pour on solution (0.5 %). For the treatment of helminth infections in horses, MOX oral gel (2 %) is currently available commercially. In early days, MOX was also shown to have anthelmintic activity and was tested as an injectable solution in horses (Lyons *et al*., 1992), but was not commercialized. This is mostly due to an unfavorable experience with ivermectin (IVM), since parenteral administration of this drug has been shown to cause adverse reactions in horses. The most frequently reported problems have included the development of local abscesses, transient midventral edema associated with death of *Onchocerca* microfilariae, swelling at the injection site and anaphylactic reactions have also been observed in a number of cases (Barragry, 1987). However, in other studies the injectable MOX did not caused any adverse reactions of toxicosis in horses (Lyons *et al*., 1992) or donkeys (Fangama *et al*., 2013).

Nowadays, the information on residual efficacy and safety of ML’s after parenteral administration in horses is still largely scarce. Despite of some risks after parenteral use of MLs in horses, the extra-label use of MOX injectable solution registered for cattle is widely used in some countries by veterinary practitioners. As one of the explanations for such a use in horses was mostly attributed to a lower cost of such treatment as compared to products registered for horses and easy use of injectable formulation with lower dose (Matthee, 2003). However, the information on the extra-label use of injectable MOX for equids and its efficacy at different dose rates was studied in a limited number of studies (Lyons *et al*., 1992; Matthee, 2003; Fangama *et al*., 2013) and requires further examination.

Reports from recent studies emphasize the growing anthelmintic resistance in cyathostomins (Matthews, 2014). Two parameters are usually assessed to measure the change in resistance state: the fecal egg count reduction and the strongyle egg reappearance period. While reduction in fecal egg count is used most frequently to assess the efficacy of all anthelmintics, the evaluation of egg reappearance period (ERP) is one of the most reliable early markers of anthelmintic resistance to MOX (Sangster, 2001) and other ML’s. In early days the ERP after treatment with MOX was evaluated to be more than 13 weeks (Jacobs *et al*., 1995) or up to 24 weeks (Boersma *et al*., 1998), indicating a long-lasting anthelmintic effect. After several years of being on the market, a study conducted in the UK showed shortened egg reappearance periods following MOX treatment to 5 – 12 weeks which were well below the values reported in the literature before (Daniels and Proudman, 2016). Moreover, numerous other studies revealed a globally widespread reduction in efficacy of MOX as assessed based on a reduced ERP (Tzelos *et al*., 2017).

The aim of present study was to examine the potency of injectable off-label used MOX solution (Cydectin 1 %; Zoetis Deutschland GmbH) and commercially available oral MOX gel for horses (Equest®; Zoetis Deutschland GmbH) to reduce the excretion of strongylid eggs in horses over a 6-week period based on fecal egg count reduction test (FECRT) and ERP.

## Material and Methods

### Animals and study design

This controlled study was carried out between February 2020 and September 2021 and was supervised by the Lithuanian University of Health Sciences. For the study, two horse farms in the bordered villages of Schweinschied and Löllbach the federal state of Rhineland-Palatinate, Germany were selected.

During the aforementioned period 28 adult horses were tested using a McMaster technique (Roepstorff & Nansen, 1998) with modifications described below for the presence of intestinal helminth infections with the owners’ consent. The horses naturally infected with strongylids and excreting a minimum number of 500 strongylid eggs per gram of feces (EPG) to yield a more accurate measurement of efficacy (Vidyashankar *et al*., 2007), having the daily access to contaminated pasture and the owners consented to handling of animals and administration of the anthelmintic drug as well as repeated rectal sampling were included into present study.

All engaged horses were warm-blooded equines of Hanoverian, Zweibrücker and crossbreeds like Anglo-Arabian and Appaloosa-Shagya Arabian breeds. Before the study, all horses were dewormed strategically with ivermectin/praziquantel oral gel when stabling in and in spring before moving out on to the pastures during at least of last 3 years.

All horses were grazing rotationally on comparable pastures during the study. The grazing pressure was on average 1.34 horses per hectare for both groups (range: 1.23 – 1.46 horses per hectare). In total each group was grazing on four different pastures rotated on average three times per grazing season from April till November. All these pastures have been grazed by horses since at least five years.

The comparable horses were allocated into two treatment groups on two farms i.e. Group IT (Intramuscular Treatment) and Group OT (Oral Treatment) according to the EPG results, the age, gender and weight, representing 83.3 % and 62.5 % of all horses on the farm, respectively. In each treatment group a comparable number of adult female and male horses were included. The horses in Group IT (N=4) were on average 16.2 years old (range: 3 – 24 years old) while those in Group OT (N=5) were on average of 13 years old (range: 3 – 23 years old).

### Treatment with moxidectin

The horses in oral treatment Group OT were treated with oral gel (Equest® Orales Gel für Pferde ad us. vet; Zoetis Deutschland GmbH) according to a manufacturer’s instructions at the dosage of 0.4 mg of MOX per kilogram of body weight (b.w.). The horses in intramuscular treatment Group IT were treated with an injectable solution (Cydectin 1 % Injektionslösung für Rinder; Zoetis Deutschland GmbH) with a concentration of 1 % at the dosage of 0.2 mg/kg of b.w. (Lyons *et al*., 1992). The amount of Cydectin 1 % solution required for injection was mixed with 5 ml of lidocaine hydrochloride solution (Lidocain 2 % Streuli ad us.vet.; Streuli Tiergesundheit AG) into one syringe at the dose of 100 mg per injection and injected under the subcutaneous tissue next to the left pectoral muscle to counteract possible side effects due to its anti-inflammatory effect (Hollmann *et al*., 2000).

### Collection and examination of fecal samples

The individual fecal samples were collected rectally from selected horses on 0, 17, 28, 35- and 42-days post treatment (d.p.t.). Rectal samples were taken twice a day, in the morning and in the afternoon and examined using a modified McMaster technique (Roepstorff & Nansen, 1998) with modifications. From each sample 4 g of feces were resuspended in 56 ml of tap water (14 ml of water per 1 g of feces), carefully mixed, filtered through the gauze, the filtrate stirred and a subsample of 10 ml poured into a centrifuge tube. After centrifugation for 7 min at 1200 rpm, the supernatant was discarded. The tube was filled with magnesium sulphate solution (density of 1.24 g/ml) up to 4 ml. The sediment was thoroughly mixed with a Pasteur pipette. Two of McMaster chambered slides were filled with a fecal suspension for each sample and total number of small strongylid eggs under four grids of McMaster chambers was then multiplied by ten resulting in a sensitivity of 10 eggs per gram of feces. Fecal samples have been microscopically examined on the same day after collection strongylid eggs identified according to the morphometric characteristics (Thienpont *et al*., 1990). The pre-treatment pooled fecal samples were collected from both treatment groups. The proportion of strongylid nematodes was examined by incubation of coprocultures, isolation of L3 larvae by baermanisation and identification based on their morphology as described (MAFF, 1986).

### Detection of resistance

To estimate the level of resistance in strongylids against MOX, a fecal egg count reduction test (FECRT) was performed and the mean number of eggs was evaluated on day 0 and 17 for horses in both groups. The sampling on day 17 was done later than the usual two-week period (Tzelos *et al*., 2017), due to a longer half-life for MOX as compared to those of IVM. The reduction in strongylid egg count (%) to each drug was calculated using the following formula (Coles *et al*., 1992):

((pre-treatment EPG – post-treatment EPG/pre-treatment EPG) × 100)

The potential anthelmintic resistance (AR) was considered if the percentage reduction in egg count was below 90 % on day 17 and the lower 95 % confidence limit (LCL) was below 80 % (Fisher *et al*., 2015). The mean efficacy of MOX was calculated as the mean percentage reduction in egg count based on the individual data on the level of reduction in egg number on days 17, 28, 35 and 42 as compared to those on day 0 for each horse in the group.

The egg reappearance period (ERP) was evaluated on each occasion the faecal samples were collected i.e. at approximately weekly and biweekly intervals until day 42 of study. The threshold for evaluation of ERP was set at 10 % below the mean percentage of FECR in group OT (the FECR on day 17 – 99.43 %) and group IT (the FECR on day 17 – 99.32 %) but determined at 17 days post-treatment i.e. for three days longer than the usual two-week period described (Nielsen *et al*., 2022b). In Group OT the ERP threshold level was therefore set at an 89.43 % and in Group IT at an 89.32 %.

### Statistical analysis

IBM SPSS Statistics 27 was used for the statistical analysis, while the FECRT was calculated manually for both groups. The figures were created in R-4.1.2 for Windows and the tables were generated in IBM SPSS Statistics 27. The differences in reduction of egg excretion between day 0 and day 17 within the groups were calculated using a nonparametric Wilcoxon Signed-Ranks Test for two paired samples with logarithmically transformed Log (x+10) egg counts. The differences in efficacy of MOX between two groups on days 17, 28, 35 and 42 were analyzed using a two tailed Fisher’s exact test without logarithmic transformation. The Wilson 95 % confidence interval and standard deviation were calculated. The P<0.05 considered as statistically significant.

## Ethical Approval and Informed Consent

All applicable institutional and international Guiding Principles for Biomedical Research Involving Animals were followed. The study was conducted in strict accordance with the Lithuanian animal welfare regulations (No. B1-866, 2012; XI-2271, 2012). All animal handling was carried out under the supervision of a veterinarian and animal welfarist Dr. A.W. Schwahn, Tierarztpraxis Dr. Aloys Schwahn, Meisenheim, Rhineland-Palatinate, Germany. Written informed consent for examination of animals and publication of data was obtained from animal owners on both farms included into the study.

## Results

**Fig. 1.**
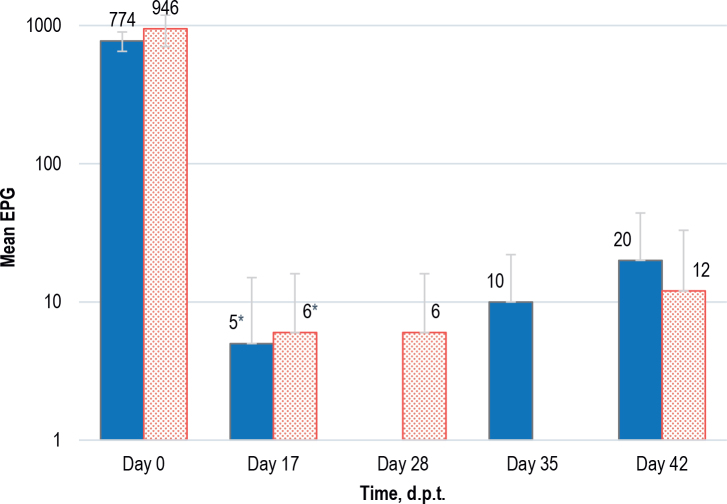
The mean number of strongylid eggs/g of feces (EPG) in Group OT (N=5; blue solid bars) and Group IT (N=4; red pattern bars) with standard deviation (SD; vertical error bars) over 42 days post treatment (d.p.t.) presented on logarithmically transformed scale. The missing values on days 28 and 35 represent 0 EPG counts. *Signifi cant reduction in Group OT (P<0.001) and Group IT (P<0.01) as compared to respective groups on day 0.

Based on the resulting data during the sampling period, a mean strongylid egg excretion in two treatment groups was estimated over a study period of 42 days (Fig. 1). The mean (M) egg excretion in Group IT has signifi cantly decreased (P<0.01) on 17 d.p.t. (M=6; SD=10) as compared to those on day 0 (M=946; SD=242). Similarly, in Group OT the decrease in mean egg excretion was signifi cant (P<0.001) on 17 d.p.t. (M=5; SD=10) as compared to those on day 0 (M=774; SD=124). Accordingly, the comparable EPG levels were recorded in both groups on day 17 of the study. From then onwards the horses in both groups had a low and comparable mean strongylid egg excretion ranging between 6 and 20 EPG on day 28, 35 and 42 that did not surpass the 10 % from the mean FEC of day 0. The identifi cation of L3 larvae in pre-treatment fecal samples revealed that the cyathostomin were the most prevalent with an average of 93 % and 95 % in OT and IT groups, respectively. Other nematode eggs observed in pre-treatment samples in some examined horses were large strongyle, *Trichostrongylus axei, Triodontophorus* spp. and *Parascaris equorum*.

**Fig. 2.**
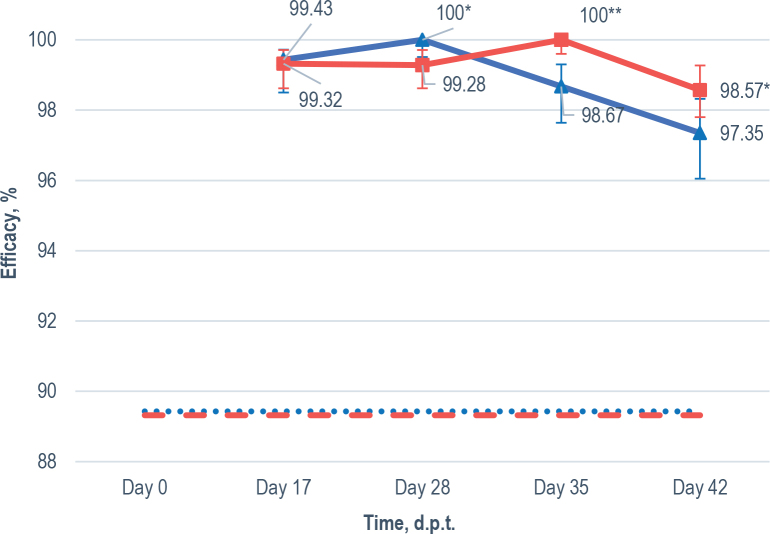
The mean efficacy of moxidectin oral gel in Group OT (N=5; blue line, bold triangles) and injectable moxidectin in Group IT (N=4; red line, bold rectangles) in reducing strongylid egg excretion and 95 % confidence intervals (vertical error bars) over 42 days post treatment (d.p.t.) with the dotted (89.43 %; Group OT) and dashed (89.32 %; Group IT) lines representing threshold set 10 % below the FECT determined at 17 days post-treatment. Significantly higher (^*^P<0.05; ^**^0.001) as compared to other group on the same time point.

The mean effi cacy of MOX in horses of both groups was continuously high over the 17 to 42 days period (Fig. 2). In Group IT (99.32 %; 95 % CI 97.62-99.71) and Group OT (99.43 %; 95 % CI 98.5-99.72) the mean effi cacy of MOX was comparable (P>0.05) on 17 d.p.t. Whereas on 28 d.p.t. it increased slightly in Group OT (100.0 %; 95 % CI 99.51-100.0) resulting in signifi cant difference (P<0.05) as compared to Group IT (99.28 %; 95 % CI 98.6299.71). From then onwards, the mean effi cacy has increased in Group IT (100.0 %; 95 % CI 99.6-100.0) and a difference became signifi cant (P<0.001) as compared to Group OT (98.67 %; 95 % CI 97.64-99.3) on day 35. Despite of decreasing effi cacy in both groups, the mean effi cacy in Group IT (98.57 %; 95 % CI 97.899.27) remained higher (P<0.05) as compared to those in Group OT (97.35 %; 95 % CI 96.05-98.32) on 42 d.p.t.

Furthermore, the anthelmintic resistance (AR) of strongylids to MOX were estimated based on FECRT results on day 17. A significant decrease in mean number of strongylid EPG from 774 to 5 (Group OT; P<0.0001) and from 946 to 6 (Group IT; P<0.003) was recorded on 17 d.p.t. (Fig. 1). The effi cacy of oral gel (99.43 %; 95 % CI 98.57-99.72) and injectable MOX (99.32 %; 95 % CI 98.62-99.71) formulations was therefore high and comparable and no resistance of strongylids to MOX was recorded (Fig. 2).

## Discussion

The MLs are the most widely used class of anthelmintics for control of nematode infections in horses around the world (reviewed by Nielsen *et al*., 2022a). Despite of substantial reliance on IVM and MOX in control of nematode infections in horses, the reports on detection of resistance to these compounds based on FECRT were relatively infrequent over the last few decades (Von Samson-Himmelstjerna *et al*., 2007; Nielsen *et al*., 2022a). The MOX is characterized by a persistent activity lasting for around 2 – 3 weeks (Vercruysse *et al*., 1998) and the efficacy against mucosal and luminal L4 stages of cyathostomes. The longer residual efficacy of MOX in horses leads to a longer exposure of parasites to this drug. This makes the efficacy longer which is reflected on the longer egg reappearance interval following the treatment (Cobb & Boeckh, 2009). Measuring the reduction in FEC at 14 – 17 days after the treatment with MOX may not therefore proof sensitive enough in evaluation of developing resistance. Though, measuring the egg reappearance period (ERP) following the treatment with ML’s may provide an early indication on reducing efficacy of the drug (Sangster, 2001). The importance of ERP detection was observed in an earlier study, where a shortened ERP in cyathostomins of less than 5 weeks following the treatment with IVM has been reported, even though it was shown to be efficious at 14 days following the treatment (Von Samson-Himmelstjerna *et al*., 2007). Similarly, in a recent study (Nielsen *et al*., 2022a) the ERP in cyathostomins shortened to 5 weeks following the treatment with IVM and MOX was recorded, while efficacy of both drugs against adults at 2 weeks post treatment was 99.9 % and 99.8 %, respectively. Thus, the reduced ERP represents a significant change in anthelmintic performance and should be monitored at weekly or biweekly intervals up to 3 – 4 months post-treatment, based on expected residual activity of anthelmintic under evaluation (Nielsen *et al*., 2022b).

In an earlier study the long egg reappearance period of up to 24 weeks after the treatment with MOX, indicating a strong anthelmintic effect was recorded in horses (Boersma *et al*., 1998). However, after 20 years of use the ERP shortened to 5 – 12 weeks following MOX treatment was documented in the UK (Daniels & Proudman, 2016) and were much below the values reported in early days. Similarly, when MOX was first introduced in US in 1997, the suppression of small strongyle egg excretion was recorded for as long as 12 – 22 weeks (Demeulenaere *et al*., 1997; DiPietro *et al*., 1997). However, a decade ago the ERP shortened to around 5 weeks (Lyons *et al*., 2011) following MOX treatment has been observed in horses. Moreover, the reduced efficacy of MOX, measured as a reduced ERP, has been observed in many other geographically widespread populations during the last decades (reviewed in Abbas *et al*., 2021; Matthews, 2014; Nielsen *et al*., 2022a). Such findings give the evidence on a shift in populations susceptibility towards the resistance to MOX and suggests that the long-lasting efficacy of MOX should be monitored following the ERP for at least of 5 weeks. The results from present study demonstrates that both formulations were comparably effective in reducing strongylid egg excretion for at least of 6 weeks with slightly reducing efficacy towards the end of examination period. Nevertheless, this reduction was not significant and suggests that in order to obtain more consistent data on duration of residual efficacy of MOX the ERP should be observed for even longer period. In case of oral administration, the lower systemic availability and shorter persistence of ML’s was usually observed as compared to those of subcutaneous administration. The subcutaneous administration tends to provide the highest availability of the drug in most animal species studied and for most of MLs and therefore should be expected to deliver the superior antiparasitic efficacy. In horses, the parenteral administration of IVM and doramectin (DOR) have been investigated (reviewed in McKellar & Gokbulut, 2012). It was shown that the output of strongylid eggs was suppressed for approximately 2 weeks longer after injectable administration of IVM to horses as compared to oral administration (French *et al*., 1983). It was anticipated that extra-label treatment with injectable MOX in present study may provide a longer lasting anthelmintic effect and/or better efficacy results. Indeed, the efficacy of injectable MOX in present study was higher starting from day 35 (P<0.001) and remained so on day 42 (P<0.05) as compared to those of oral gel treatment. Despite of slight increase in egg excretion in both, oral and injectable treatment groups, the efficacy of injectable MOX still remained at the higher level (98.57 %) as compared to those of oral gel (97.35 %) on day 42 post treatment.

In early days the efficacy of injectable MOX was tested in a series of critical studies (Lyons *et al*., 1992). It was shown that MOX was comparably effective in reducing the number of strongyle and small strongyle eggs in feces at the dose of 0.2 mg/kg^−1^ by 99 % and 97 %, and at the dose of 0.4 mg/kg^−1^ by >99 % and 97 %, respectively. The recovery of *Strongylus vulgaris* (0.2 mg/kg^−1^) and *S. vulgaris*/*S. edentatus* (0.4 mg/kg^−1^) was reduced by 100 % after the treatment. Furthermore, the efficacy of extra-label used injectable MOX registered for cattle was tested on 10 weaners in South Africa (Matthee, 2003), however, it was ineffective in controlling the cyathostomin infection. This was primarily related to a possible under dosing of the drug since 0.2 mg/kg of b.w. of injectable MOX was considered as half of the dose required for horses. In present study treating the adult horses with injectable MOX at the dose of 0.2 mg/kg of b.w. was highly effective (99.32 %) to reduce the strongylid egg excretion as compared to those of oral treatment (99.43 %) on 17 d.p.t. Furthermore, the effect of injectable treatment at twice lower dose (0.2 mg/kg of b.w.) was higher on 28 and 42 d.p.t. as compared to oral treatment (0.4 mg/kg of b.w.). The results of present study give an indication on the more persistent anthelmintic effect of injectable versus oral MOX, which further should be tested with incorporation of pharmacological parameters in more extended studies.

The anti-endotoxic and anti-inflammatory effect of systemically administered lidocaine is documented in a number of studies (Mudge, 2007). It is believed that lidocaine affects a complex of inflammatory processes such as phagocytosis, exocytosis, migration and cellular metabolism. However, the mechanism for its anti-inflammatory effect is not fully understood (Karnina *et al*., 2021). In present study, the role of lidocaine in preventing local side reactions after MOX injection could not be evaluated due to the absence of control group without lidocaine. However, the fact that no side effects (local abscesses or swelling) developed at the site of injection and no other side effects were recorded in horses treated with injectable MOX does not excludes an anti-inflammatory effect of lidocaine administration. While the risks and mechanism of possible side reactions caused by injectable ML’s is not evaluated in horses, lidocaine may be considered as a candidate compound in reducing local and/or other inflammatory reactions in cases of off-label used ML’s. In present study the lidocaine was mixed together with moxidectin solution into one syringe at the rate of 5 ml of lidocaine 2 % solution (i.e. 100 mg per injection) with 8 – 12 ml of Cydectin, amounting to a 0.59 – 0.77 % lidocaine concentration for subcutaneous injection. However, the reference dose, concentration for lidocaine solution and its anti-inflammatory effect in preventing possible side effects caused by injectable ML’s should be tested in more extended studies.

In present study no resistance of strongylids to MOX was detected. On both farms the horses excreting the high number of strongylid eggs (EPG over 500) were selected for the study representing the majority of horses on the farm. Moreover, variability between two groups was kept as low as possible by attempt to include the horses with comparable age, weight and sexes into both groups. Consequently, it was expected that high enough EPG counts and limited their variation in both groups allowed the accurate enough measurement of resistance and therefore the resistance of strongylids to MOX, if present in this study, would not go undetected. The results from present study gives the evidence that injectable MOX in case of extra-label use in adult horses at the dose of 0.2 mg/kg of b.w. was able to reduce the excretion of strongylid eggs in feces effectively for at least of 6 weeks and was safe to use in horses. However, to fully test the efficacy and early indication of the shift in strongylid populations susceptibility towards the resistance the examination of ERP should be continued for more than 6 weeks.
